# A needs assessment for enhancing workplace-based assessment: a grounded theory study

**DOI:** 10.1186/s12909-024-05636-3

**Published:** 2024-06-13

**Authors:** Vasiliki Andreou, Sanne Peters, Jan Eggermont, Birgitte Schoenmakers

**Affiliations:** 1 Academic Centre for General Practice, Department of Public Health and Primary Care, KU Leuven, Leuven, Belgium; 2 School of Health Sciences, Faculty of Medicine, Dentistry and Health Sciences, The University of Melbourne, Melbourne, Australia; 3 Department of Cellular and Molecular Medicine, KU Leuven, Leuven, Belgium

**Keywords:** Medical education, General practice, Workplace-based assessment

## Abstract

**Objectives:**

Workplace-based assessment (WBA) has been vigorously criticized for not fulfilling its educational purpose by medical educators. A comprehensive exploration of stakeholders’ needs regarding WBA is essential to optimize its implementation in clinical practice.

**Method:**

Three homogeneous focus groups were conducted with three groups of stakeholders: General Practitioner (GP) trainees, GP trainers, and GP tutors. Due to COVID-19 measures, we opted for an online asynchronous form to enable participation. An constructivist grounded theory approach was used to employ this study and allow the identification of stakeholders’ needs for using WBA.

**Results:**

Three core needs for WBA were identified in the analysis. Within GP Training, stakeholders found WBA essential, primarily, for establishing learning goals, secondarily, for assessment purposes, and, lastly, for providing or receiving feedback.

**Conclusion:**

All stakeholders perceive WBA as valuable when it fosters learning. The identified needs were notably influenced by agency, trust, availability, and mutual understanding. These were facilitating factors influencing needs for WBA. Embracing these insights can significantly illuminate the landscape of workplace learning culture for clinical educators and guide a successful implementation of WBA.

## Introduction

In medical education, the need for accountability and for reassurance of competence should make learning and assessment inextricably linked [1]. Good assessment of clinical competence affects and involves multiple stakeholders, not only core stakeholders, such as trainees^1^, trainers, and universities, but also broader, other health professionals, policy makers, and, most importantly, patients and general public [2]. Good assessment models should safeguard and guarantee patients’ safety as well as providing contextual and on-the-spot feedback to trainees [3, 4]. 

A new approach of assessment tailored to authentic workplace-based environments has emerged as to provide higher accountability and high quality of patients’ care [5]. Workplace-based assessment (WBA) refers to the assessment of physicians’ actual performance carried out independently in professional practice. In an educational context, WBA implies gathering data about trainees’ performance in an authentic context and providing relevant feedback for further improvement. The literature illustrates a number of existing WBA instruments, which aim to assess various aspects of competence (mini–Clinical Evaluation Exercise, Case-based Discussion, Direct Observation of Procedural Skills, Multisource Feedback, etc.) [6]. These instruments aim, on the one hand, at evaluating trainees in authentic assessment environments, and, on the other hand, at providing feedback and fostering reflective practice.

Although WBA has seen extensive implementation, its educational value has been questioned in postgraduate medical education [7]. Prevailing perception among stakeholders often leans towards negativity, labelling WBA as a bureaucratic burden. This adverse outlook is rooted in several key factors, including lack of time, poor trainers’ engagement, misunderstanding of WBA objectives, and inadequate feedback quality [8]. However, research has mainly focused on exploring stakeholders’ perceptions about WBA, largely disregarding the vital aspect of stakeholders’ educational needs in clinical practice [9]. By employing a needs assessment framework, we can identify implementation facilitators and barriers, and, subsequently, enhance implementation success. Therefore, the aim of this study was to explore what stakeholders need from WBA, and to identify factors that hinder or foster these needs in the context of a General Practitioner (GP) Training Program.

## Methods

### Educational context

This study focused on WBA activities embedded in the postgraduate GP Training in Flanders, Belgium. The GP Training is a 3-year postgraduate curriculum, where trainees must take a series of WBAs during their two GP internships, which last 12- and 18-months respectively. All WBAs should be registered and documented in an electronic (e-) portfolio available for all the stakeholders involved. At the clinical workplace, trainees closely work with workplace-based trainers. These trainers are experienced GPs who mentor trainees in their own practice. To become trainers, they must have completed specific educational programs, such as courses on providing feedback, guiding daily practice, and assessing trainees. They are responsible for providing hands-on, practical training and sharing their day-to day experiences with their trainees. Their duties include teaching and showcasing necessary knowledge, skills, and attitudes to their trainees. They offer direct observation and guidance, provide feedback, and facilitate the learning process at the clinical workplace. According to the official assessment regulations of the GP training, trainers have to perform at least 5 mini-clinical evaluation exercises with their trainees within an academic year, either by directly observing them performing clinical work or through videorecorded clinical encounters [10]. Additionally, trainers should annually perform 3 evaluations of clinical events in which trainees receive feedback on different competency domains, specifically based on the Canadian Medical Education Directions for Specialists framework (CanMEDS) [6, 11]. Also, trainees often have case-based discussions with their trainers on a daily and weekly basis. Trainees present a patient case to their trainers who critically evaluate the decision-making procedure, communication with the patient, and consultation management.

Besides the workplace-based trainers, university-based tutors, who are compensated for their roles and have received specialized training, also support trainees in groups. The aim of these groups is to provide peer feedback and support to the trainees biweekly. Tutors are also experienced GPs and affiliated with the collaborating universities. They are usually involved in overseeing and ensuring trainees’ progress during clinical internships. Tutors are predominantly responsible for ensuring that the training objectives are met within each but also across various internships. Although they may not be involved in direct day-to-day training, they play a crucial role in WBAs. Tutors must conduct case-based discussions with trainees, where trainees present patient cases illustrating clinical decision making, clinical reasoning, communication skills, and diagnostic skills. Also, tutors should enhance multi-source feedback, by promoting peer-feedback on the videorecorded encounters, and by compiling feedback information and sources, including trainees’ e-portfolio, to identify areas for improvement. There is officially no stipulated minimum number of WBAs that tutors should perform with their trainees. Nevertheless, the compiled feedback should be discussed at least 3 times per academic year between tutor and trainee.

### Participants

Three different groups of stakeholders participated in this study: GP trainees, GP trainers, and GP tutors. All participants had at least one year of experience with WBA. Trainees were either at the end of the second or the third year of the GP Training. To explore the needs of the different groups, purposeful sampling was used to recruit participants [12]. First, the lead investigator invited tutors by email. Afterwards, trainees were recruited through an open call via social media groups. Lastly, trainers were invited by the principal investigator and member of the research team.

### Study design

We employed a qualitative study design following a constructivist grounded theory approach [13]. We chose this approach to delve into the subjective experiences of trainees, trainers, and tutors regarding WBA. Constructivist grounded theory recognizes that knowledge is co-constructed between interviewee and interviewer, allowing a deeper understanding of individual and contextual influences on participants’ perspectives [13]. We conducted online asynchronous focus groups as they encourage interaction among the participants [14]. The online design was preferred because of the measures against the spreading of COVID-19. To facilitate participation throughout the pandemic, we chose to administer the focus groups asynchronously. The asynchronous format provided the necessary flexibility to the participants without hindering their clinical practice [15]. The stakeholders were divided into different groups based on their role in the GP Training: trainees, trainers, and tutors. We chose homogeneous instead of heterogeneous focus groups to give the freedom to the participants to express freely their opinion, and to avoid potential power relationships influencing their opinion. To facilitate the focus groups and to guarantee data richness, we set a minimum of 6 and a maximum of 8 participants per group [16]. 

### Data collection and analysis

To achieve methodological rigour, we developed an interview guide with main and supplementary questions, ensuring a logical and thorough investigation of participants’ needs about WBA. Initially, we engaged in discussions as a research team. We are a team of four researchers, consisting of two researchers with a background in education and two researchers with a medical background. After establishing a thorough understanding of the context, we created an analytical diagram, structuring the main and the supplementary questions to ensure that the data collection was systematic and logical. All questions were open-ended in order to capture the depth and breadth of participants’ needs on WBA. This guide was iteratively refined after each focus groups, based on ongoing analysis, aligning with the constructivist grounded theory approach [13]. The main questions were open-ended and focused on WBA: (a) What does WBA mean for you?, (b) How would you describe WBA?, (c) For which purposes would you use WBA?. To collect our data, a researcher guided the focus groups, while the principal investigator, a GP and clinical teacher, participated as observer to monitor the process. To ensure consistency of data collection, three members of the research team discussed the different procedures before each focus group.

The data were collected using an online software called FocusGroupIt [17]. This tool provided the opportunity for the participants to participate anonymously. Anonymity allowed an increased ease to discuss recurring problems of WBA [15]. Before starting, participants received an e-mail invitation to register to the platform. They could choose their own pseudonym that was seen by other participants. The participants’ real names were only visible to the moderator. The focus groups lasted from 2 to 3 weeks each. Questions were posted online by the moderator, while enough time was given to the participants (approximately 3 days per set of questions) to respond to the questions and interact with each other. Reminders were sent if the moderator thought it was necessary. When more clarifications were required, sub-questions were posted to delve more in depth and elucidate participants’ reactions. Data collection and data analysis took place between June 2020 and October 2020.

To analyse the data, we used the Qualitative Analysis Guide of Leuven (QUAGOL) [18]. The coding process was done by two researchers separately [19]. Discrepancies in coding were discussed until consensus was reached, and a third researcher was advised, when necessary. For data analysis, we used NVivo QSR International (Release 1.0). Following constructivist grounded theory, memos were firstly written before the coding started [13]. The coding process happened in three phases. During initial coding, we focused on small units of analysis, coding line-by-line. During focused coding, we focused on frequent earlier codes to navigate through the data, and we discerned initial codes with the most analytical strength [13]. During axial coding, we focused on relations between categories and subcategories of codes [13]. 

## Results

**Fig. 1:**
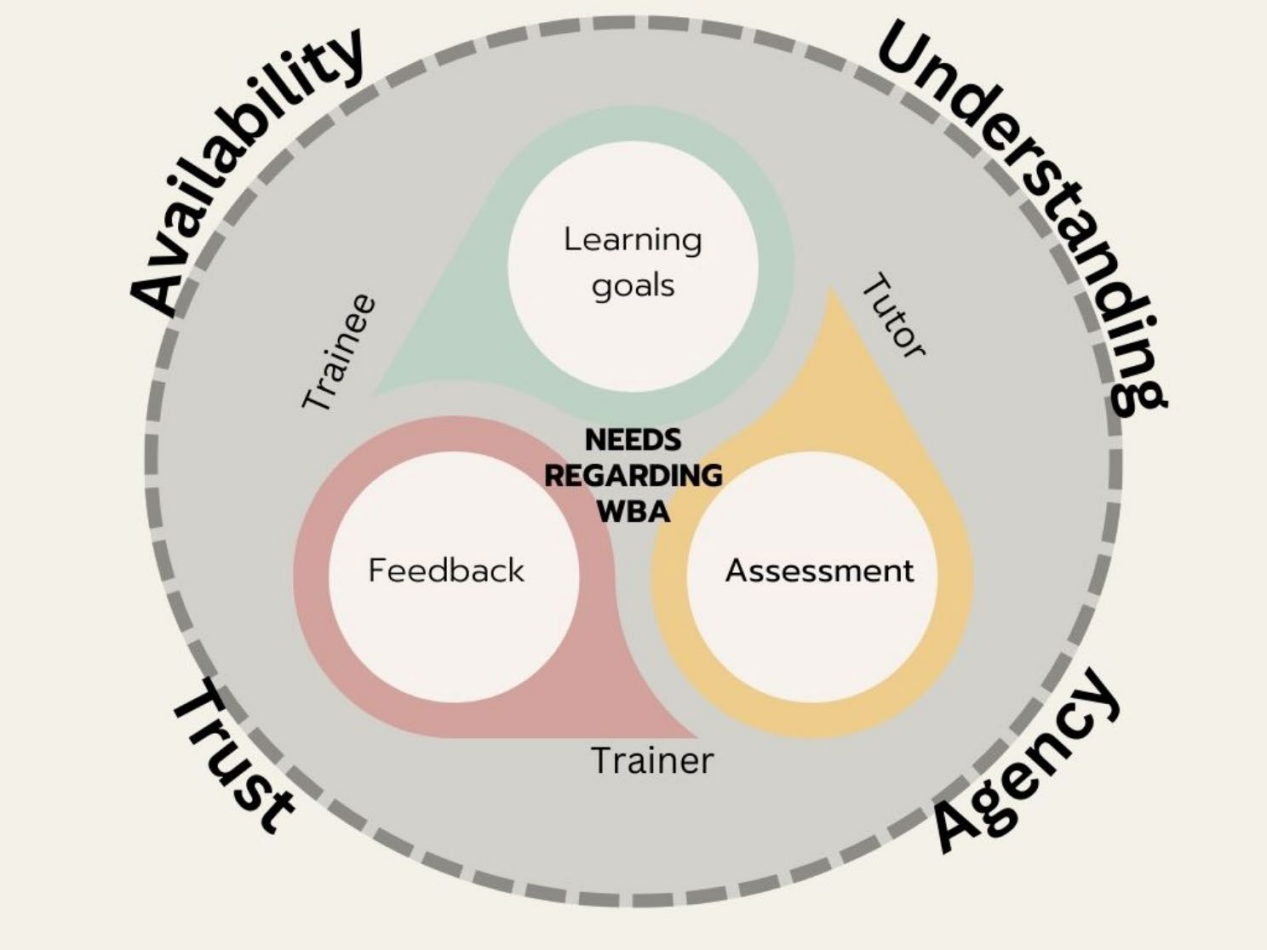
.

Three online asynchronous focus groups(*N* = 3) were conducted, one with trainees (*n* = 6), one with trainers (*n* = 7), and one with tutors (*n* = 8). The results showed that trainees, trainers, and tutors need WBA to fulfil three different educational needs: (1) need for establishing learning goals, (2) to construct an idea of trainees’ clinical competence, (3) to give or receive feedback. There was no hierarchical order among these three needs, but they rather displayed a continuous circular relationship. That implies that they alternated depending on the context and educational purpose. Also, these needs seemed to be influenced by four different factors, namely availability of trainers and tutors, mutual understanding, trainees’ agency, and trust between trainer and trainee. These factors seemed to facilitate and augment the educational value of WBA. Figure 1 illustrates the three needs and the important factors influencing them along the three stakeholders’ groups. In the following section, these different needs and the different factors are presented with examples of verbatim quotes.

1. Need for establishing learning goals.

Consensus among all participants affirmed the necessity for WBA to effectively support and enable trainees in establishing their learning goals. Trainees emphasized the importance of WBAs enabling them to proactively participate in defining their personal learning goals, as they felt as primarily responsible for shaping their learning process. Through WBA, trainees expressed their need to feel in control of their learning. For trainees, WBA should provide enough performance evidence in order to define their further learning agenda. This sentiment was echoed by both trainers and tutors, who underlined that the learning and delineating the learning trajectory rested squarely on the trainees themselves.

Of course I expect a good trainee to engage in self-reflection after an (workplace-based) evaluation. A trainee has to formulate learning goals based on this feedback and actively act further on it. My job (as tutor) is to follow-up these goals in the long run, compare them with other trainees, and help with the overall functioning of the trainee as a doctor in clinical practice (GP tutor 2).

I formulated my learning goals for this internship at the beginning, after a couple of (workplace) evaluations in the (GP) practice where I am now, and I have adjusted them along the way. I formulate them based on difficulties I experience myself at the workplace, feedback from my trainer or my tutor after evaluations (GP trainee 6).

However, some trainees found this task troublesome. They expressed the need for support after WBAs in order to be able to be in charge of their learning development. Specifically, they admitted that formulating efficient learning goals was a skill that needs to be trained and exercised.

My learning goals (for my traineeship) in the beginning were pretty vague and not concrete enough, and were much less helpful in directing my learning. Only in my second year -at the request of my trainer -I started to formulate my learning objectives SMART(Specific, Measurable, Attainable, Relevant, and Time-bound) and became more successful in planning my learning process (GP trainee 2).

The frequency of WBA posed significant obstacles to this need, as reported by trainees. Several trainees expressed their concern that WBA activities lacked a systematic and regular schedule, making it difficult for them to maintain a consistent pace in their learning.

I do not have that feeling (of getting help) because assessment moments with my trainer do not happen frequently enough (GP trainee 1).

Learning to set up learning objectives accurately and specifically, and to follow up regularly with the trainer, is, in my opinion, a must in an educational setting. Whether this actually happens now still depends on who your trainer is and whether they consider this important, which all too often is not the case (GP trainee 3).

Moreover, trainees faced an additional challenge due to the absence of a well-defined learning plan for their training. This lack of guidance led to frustration as trainees remained uncertain about the adequacy of their learning and professional development.

There is

no listing of specific learning objectives to be achieved or concrete topics within the different CanMEDS roles (GP trainee 6).

2. Need for assessing or being assessed.

Most importantly, WBA was conceived by all the stakeholders as a necessary assessment to get insights into trainees’ clinical competence. Provided consistency, it could yield a holistic representation of trainees’ performance. Nevertheless, trainees’ perspectives about the essence of clinical competence were diverging from those of trainers and tutors.

For trainees, clinical competence encompassed the practical aspects of functioning as a doctor on a day-to-day basis. In this context, WBA played a crucial role in identifying their specific learning needs. Trainees used phrases such as “to help you further”, and “daily evaluation moment to support the learning process” when articulating their requirements for WBAs.

For trainers and tutors, WBA was necessary to foster continuous learning and developmental progression. This continuity of evaluation would provide a better idea of how trainees function as doctors and aimed at enhancing learning growth by giving direct feedback linked to actual performance at the workplace. By using WBA instruments frequently, trainers believed that they would detect gaps in trainees’ clinical performance:

In my opinion, this means a more continuous evaluation…

where the daily function in the clinical practice is closely observed (GP trainer 1).

3. Need for giving or receiving feedback.

WBA was also conceived necessary for streamlining the feedback process. All participants valued the feedback opportunities that it provided. Each group put emphasis on different facets of the feedback process. In particular, trainees highlighted the comprehensive nature of feedback following WBAs, which could contribute to their development by fostering self-reflection on their performance. Trainees explained that feedback should contain concrete steps on how to improve their performance, and, eventually be linked to on patient-cases.

Most feedback should not be offered during official feedback moments, but in-between, during daily meetings about specific (patients) cases (GP trainee 5.

Also, creating a conducive environment for feedback after WBAs was crucial for the trainees, with a key prerequisite feeling safe within their relationship with their trainer. A foundation of trust between trainee and trainer could elevate trainees’ engagement, supporting a safe environment where they felt comfortable seeking clarification and engaging in discussions. Additionally, the opportunity for active participation in shaping their own feedback was underscored as highly significant. Trainees agreed that being able to play a proactive and constructive role in the feedback process could enhance their engagement in the assessment process.

That (being able to participate in the feedback process) is highly dependent on the GP practice. In the practice where I did my first-year traineeship, there was a very hierarchical relationship (between me and my trainer) and during (our) discussions it was discussed mainly what my trainer wanted to and most often that was only one thing, which he did not like, so all the rest was not mentioned. In the practice where I am now, we are conceived as equals. Everything is open for discussion, everyone is flexible. The trainer gives (me) feedback without imposing things on me (GP trainee 3).

Trainers and tutors also elaborated on the condition of trust in their relationship with their trainees. They perceived it as their role to establish this security feeling in their relationship. Most trainers and tutors mentioned that this trust relationship was a necessary component of a learning culture within their clinical practice. A relationship based on mutual trust also facilitated giving negative feedback. The more open this relationship was, the easier it was for trainers and tutors to discuss mistakes detected in clinical practice during a feedback moment.

## Discussion

This study aimed at exploring stakeholders’ needs regarding WBA in a postgraduate GP Training. The findings unveiled a triad of educational needs that GP trainees, GP trainers, and GP tutors wished to address through WBA. Notably, these needs do not solely relate to assessment of clinical competence, but encompass a broader scope that includes workplace learning and culture. Which need predominates in WBA hinges on the specific learning requirements of the trainees at that moment.

In this study, WBA needs were discussed from different standpoints to explore underlying foundations. As assessment involves different stakeholders, incorporating different perspectives allows a deeper understanding of the common ground. Overarching requirements could assist to refine WBA practices and to shed light on the interplay between learning culture and assessment activities.

A shared need across all the three focus groups was that WBA is needed to support establishing learning goals. Particularly among trainees, the value of WBA was emphasized as a potent tool for shaping and refining learning goals tailored to their unique clinical contexts. Nevertheless, trainees experienced the lack of well-defined learning plan for their clinical practice as an obstacle. This hurdle underscores the impetus towards embracing competency-based medical education [20]. Shifting towards a competency-based assessment framework within the workplace would empower trainees to progress through the program by progressively showcasing specific competencies. Defined outcomes would provide a structured framework for WBA, outlining essentials competencies trainees must attain at different levels of their training [21]. 

Additionally, availability, trust, and mutual understanding among the stakeholders were also highlighted in this study. Within the trainer-trainee dynamic, these three factors emerged as catalysts for the organic integration of WBA in clinical practice. Especially, trust seemed to be a pivotal factor within the trainer-trainee relationship. Our findings align with other qualitative studies about how perceived trust influences WBA [22]. This personal trust facilitated, on the one hand, trainees to require more assessment by the trainers, and, on the other hand, trainers to provide more meaningful and, most importantly, negative feedback, when necessary.

This study also found that student agency influenced how trainees view WBA. Trainees were keener on using WBA, when they felt that they could constructively contribute to the assessment and feedback process. The importance of agentic engagement in the learning process has recently been demonstrated in the literature as well [23, 24]. Trainees overwhelmingly valued opportunities to be involved in their own evaluation either by initiating an assessment moment and by asking specific feedback or by engaging in broader conversations about medical guidelines and evidence-based medicine. The student agency in the assessment process was also acknowledged by trainers [25]. Through supportive mentorship and open guidance, trainees were encouraged to seek feedback and learn from their mistakes.

### Limitations

We acknowledge that some limitations should be considered, when interpreting our data. First, while we used a constructivist grounded theory approach, we limited the number of our focus groups to three. This limitation was a consequence of the COVID-19 pandemic. Our study population consisted of medical professionals who experienced an enormous workload depending on the varying imposed countermeasures for COVID-19. Also, because of newly imposed measures against COVID-19, five trainers decided to drop out of the study. Subsequently, we invited five new trainers to participate in the trainers’ focus group. Second, we collected a limited range of demographic data from our participants to respect the GDPR policy. This might impede a potential generalization of our study. However, the purpose of grounded theory in qualitative research is to explore, understand, and review the concepts emerged in the data, rather than making inferences to the population [13]. Third, we used only one method to gather data, while conceptions are complex, multi-dimensional, and have to be elicited [26]. Nevertheless, by applying data and investigators triangulation and including multiple perspectives, we tried to produce a more comprehensive view on which needs WBA should answer to [27]. Coding the data by two different researchers allowed the emergence of different observations and interpretations [19]. Moreover, the asynchronous format of the focus groups could hamper a more fluid discussion and interaction between participants. However, we opted for this format, since participation at a moment of convenience was more important for our study population during COVID-19 lockdowns. A last limitation of this study is that recruitment took place on voluntary basis. Consequently, stakeholders who did not participate might hold other concepts about WBA. To mitigate this selection bias, we purposefully announced the study through different communications channels for GPs, including social media, inviting various stakeholders to spontaneously contribute to the findings.

## Conclusion

The critique on WBA makes a strong case for exploring what stakeholders need for using WBA in clinical practice. The needs that emerged significantly enrich the realm of the qualitative research on WBA. Stakeholders need WBA for supporting learning development, for assessment, and for feedback purposes. These requirements do not solely relate to assessment of clinical competence, but also exhibit the influence of workplace culture on assessment. Nevertheless, further research should focus on a deeper understanding on how trust embedded in learning culture can configure and shape WBA during clinical training. Clinical rotations and dynamic clinical environment might impair WBA by inhibiting the development of trust relationships between the stakeholders. Further qualitative research is needed to explore how agentic engagement influences WBA, and how it affects trainees’ assessment practices.

## Data Availability

The datasets used and/or analysed during the current study are available from the corresponding author on reasonable request.

## Footnotes

1 In medical education literature, trainees are often referred to as residents or medical students.

## Abbreviations

WBA: Workplace-based assessment
GP: General Practitioner
e-: Electronic
CanMEDS: Canadian Medical Education Directions for Specialists
